# Differential influence of cortex and stele components on root tip diameter in different types of tropical climbing plants

**DOI:** 10.3389/fpls.2022.961214

**Published:** 2022-08-31

**Authors:** Haiwu Xu, Siyuan Wang, Liang Tang, Yan Wang, Zhongyue Li, Wenna Wang

**Affiliations:** ^1^Key Laboratory of Germplasm Resources of Tropical Special Ornamental Plants of Hainan Province, College of Forestry, Hainan University, Haikou, China; ^2^School of Forestry, Northeast Forestry University, Harbin, China; ^3^Taishan Forest Ecosystem Research Station of State Forestry Administration, State Forestry and Grassland Administration Key Laboratory of Silviculture in downstream areas of the Yellow River, College of Forestry, Shandong Agricultural University, Taian, China

**Keywords:** root tip diameter, anatomical trait, relationship, lianas, vines

## Abstract

Climbing plants are an abundant and taxonomically diverse plant group that competes intensely with trees and thus substantially affects forest diversity and structure. The growth and physiology of climbing plants largely depend on their root tip structure and function. However, little is known regarding the mechanisms through which anatomical traits regulate root tip diameter in climbing plants. Therefore, our study sought to explore the relationships between root tip diameter and seven anatomical traits (e.g., cortex thickness and stele diameter) in three lianas and three vine species sampled from a tropical forest in Hainan. Root tip diameter was significantly positively correlated with cortex thickness (*r* = 0.94–0.99) and stele diameter (*r* = 0.72–0.94) within species, especially with cortex thickness. Cortex thickness was significantly positively correlated with mean cortical cell diameter in six species (*r* = 0.72–0.93), but was only correlated with the number of cortical cell layers in three species (*r* = 0.42–0.66). Stele diameter displayed significant positive correlations with mean conduit diameter (*r* = 0.58–0.88) and the number of conduits per stele (*r* = 0.50–0.66, except for *Cyclea hypoglauca*), and was negatively correlated with conduit density in all species (*r* = −0.65 to –0.77). The correlations between cortical cells and conduit traits and root tip diameter were similar to that with cortex thickness and stele diameter, respectively. Compared with vines, liana root tips showed closer relationships between root diameter and cortex thickness and stele diameter, and between cortex thickness and mean diameter of cortical cells. Moreover, the root tip of lianas possesses significantly higher stele proportion and denser conduits, significantly lower cortex proportion, and smaller conduit size than those of vines. However, the specific conductivity was similar. Overall, these results suggest that the cortex is the main driver for the change in root tip diameter rather than the stele. Nevertheless, both factors were responsible for variations in diameter-related traits when compared with number-related traits, with lianas and vines exhibiting distinct regulatory mechanisms.

## Introduction

Climbing plants constitute an abundant and taxonomically diverse plant group that competes intensely with trees and thus substantially affect forest diversity and structure (Phillips et al., 2002; Wright et al., 2004; Schnitzer and Bongers, 2011). This is because climbing plants are capable of forming a dense carpet covering the tree canopy, accounting for approximately 20% of the total leaf area of the forest (Putz, 1983) and substantially hindering the light acquisition of individual trees (Putz, 1984; Schnitzer and Bongers, 2002; Ingwell et al., 2010). Furthermore, climbing plants experience less physiological stress than trees during seasonal drought (Smith-Martin et al., 2019), and steadily increase in abundance and biomass in the tropics (Collins et al., 2016). Therefore, they severely reduce tree growth rates, fecundity, and survival in forests (Schnitzer et al., 2005). Thus, studying the physiological and ecological functions of climbing plants would provide key insights into the dynamics of interspecific competition and vegetation composition in forest ecosystems.

The growth and physiological functions of plants largely depend on the structure and function of the root tips, which are the primary structure absorbing water and nutrients from soils (Pregitzer et al., 2002; Guo et al., 2008b). The structure and function of root tips are mainly characterized by functional traits of root tip diameter, cortex thickness and stele diameter (Guo et al., 2008b; Kong et al., 2014). Nevertheless, very few studies have investigated the functional traits of climbing plant root tips. Drawing upon the study of self-supporting plants, root diameter is a direct functional trait that reflects root physiology in herbaceous and woody species (Burton et al., 2012; Comas et al., 2012; Bowsher et al., 2016), with thicker root tips exhibiting weaker absorption but stronger transportation (Guo et al., 2008b; Jia et al., 2010). This is because the cross-sectional size of the root tip can vary depending on its anatomical structure (i.e., cortex and stele), which are important tissues responsible for the absorption and vertical transport of individual plants, respectively (Esau, 1977; Genre et al., 2008; Guo et al., 2008b; Comas et al., 2012). In the study of self-supporting plants, root tip diameter change was more strongly influenced by the variation of cortex thickness or cortical cross-sectional area rather than stele diameter in both herbaceous and woody species (Hummel et al., 2007; Gu et al., 2014a). One of the important reason is that root cortex accounts for a larger proportion in the cross-section area of root tips (Esau, 1977; Guo et al., 2008b; Gu et al., 2014a). Wang et al. (2020) studied on three temperate climbing plant species and found that cortex occupied a larger proportion of the cross-sectional area of root tip diameter than stele. It can be expected that cortex has stronger effects than stele in the regulation of root diameter in climbing plants, but this has not yet been investigated.

Additionally, few studies on climbing plants have investigated the mechanism of changes of the cortex thickness and stele diameter. In self-supporting plants, the variation of root tip cortex thickness and stele diameter are fundamentally caused by the changes in cortical cell and conduit traits (e.g., size and number) (Dong et al., 2015; Wang et al., 2018). Previous studies on self-supporting plants have reported that root physiology varied widely when different features (i.e., diameter- or number-related traits) of cortical cells and conduits were altered. For example, increasing the conduit and cortical cell diameters rather than increasing the number of conduits and cortical cell layers can more effectively improve vertical transport (Tyree and Ewers, 1991) and reduce metabolic consumption in roots (Chimungu et al., 2014), respectively. Climbing plants are characterized as the extremely wide vessels in their tall and slender stem compared to trees (Ewers et al., 1990; Hacke et al., 2000; Hu et al., 2010; Schnitzer and Bongers, 2011), therefore it appears very likely that the diameter-related traits of anatomical components (i.e., cortical cells and conduits) play more important roles than number-related traits in the regulation of cortex thickness and stele diameter, respectively, and thus contribute greatly to the variations in root tip diameter for this type of plants. However, whether this regulation exists in the root tips of climbing plants has not yet been investigated.

Lianas (woody climbing plants) and vines (herbaceous climbing plants) are two distinct types of climbing plants that show obvious differences in their life cycle, geographical distribution, and sensitivity to environmental changes (Jiménez-Castillo et al., 2006; Hu et al., 2010; Angyalossy et al., 2012). However, few studies have focused on the differences between lianas and vines in the distribution of anatomical structure and the structure–function linkage of the root tips. Drawing upon the findings of self-supporting plants, 50 tropical and temperate tree species (Gu et al., 2014a) showed a higher interspecific variation in root tip diameter than 14 herbaceous Mediterranean species (Hummel et al., 2007), while the proportion of stele diameter in root diameter was more stable and higher among the woody species (mean and range of 25.7 and 23.5% ~ 28.8%, respectively) than herbaceous species (mean and range of 21.9 and 11.8% ~ 40.1%, respectively). These results suggest that, in self-supporting plants, the association of root diameter with anatomical traits appeared stronger in woody plants than in herbaceous plants. Woody and herbaceous plants were widely accepted to be distinctly different in life cycles and evolutionary histories (Ma et al., 2018), it seems likely that the effect caused by the difference between woody and herbaceous plants on root structure and function should be stronger than those caused by the difference between climbing and self-supporting plants. However, it is still unclear whether climbing plants have similar anatomical patterns to that of self-supporting plants, i.e., root tips of woody plants (i.e., lianas in climbing plants) have a greater proportion of stele and tighter association of root diameter with anatomical traits than herbaceous plants (i.e., vines in climbing plants).

**Figure 1:**
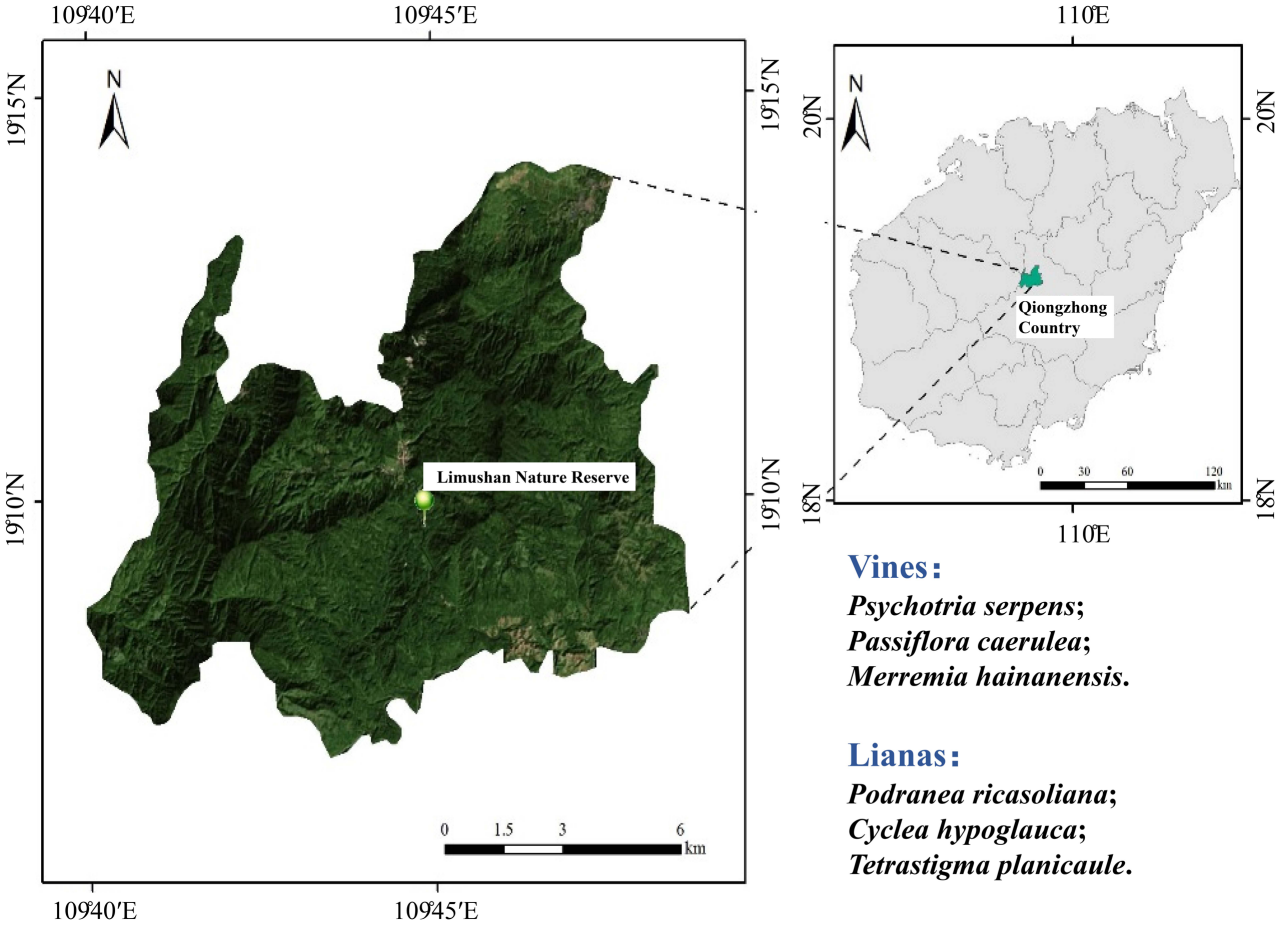
Location of Limushan Nature Reserve in Qiongzhong County, Hainan Province, China.

**Figure 2:**
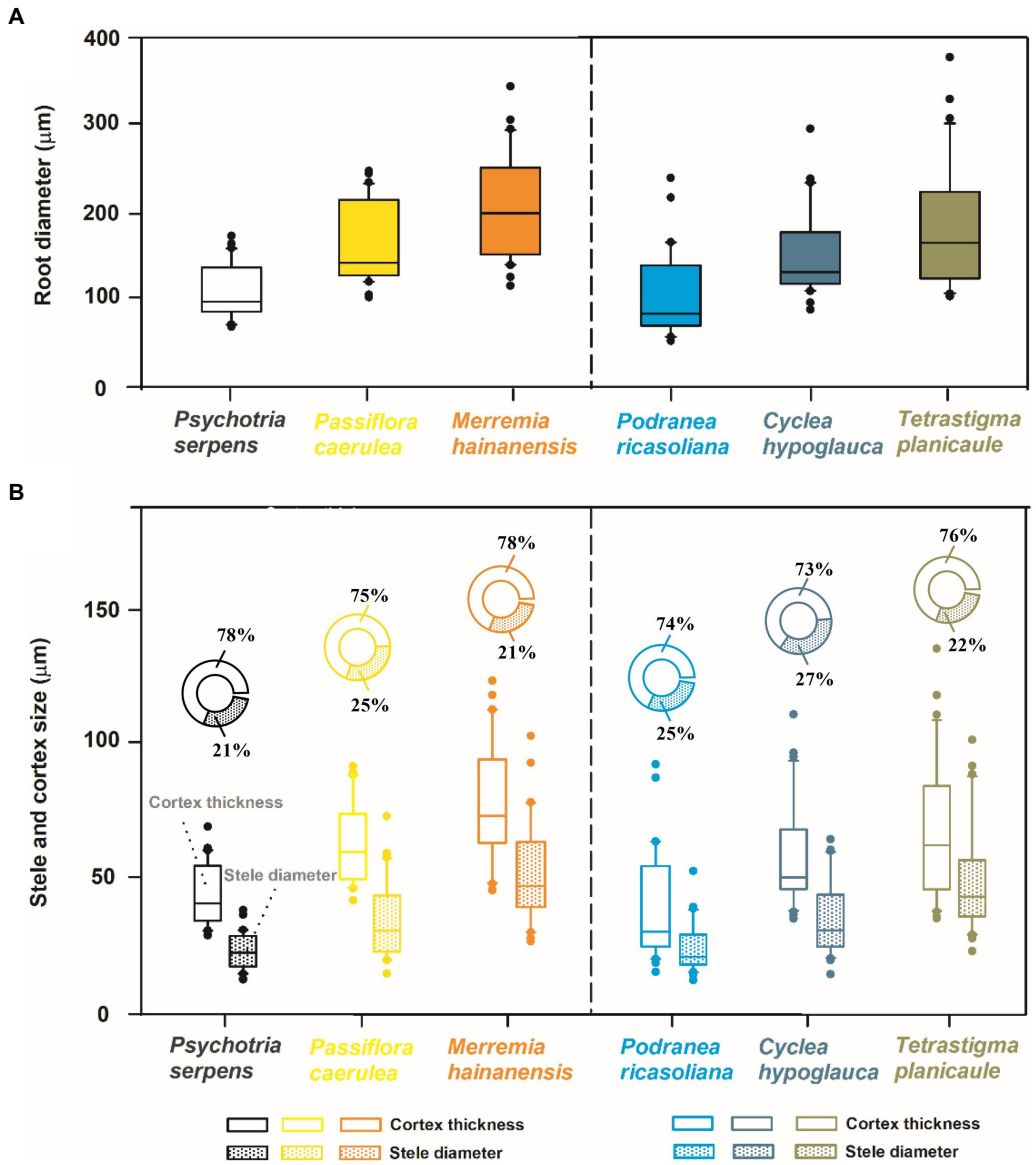
Mean values of root tip diameter **(A)**, cortex thickness, stele diameter and the proportion of these two anatomical traits to root tip diameter **(B)** in three vine (herbaceous climbing plants) and three liana (woody climbing plants) species in tropical forest (*n* = 30).

The objectives of this study were to investigate the relationships between root tip diameter and several vital anatomical traits, as well as to explore the differences in these intraspecific relationships and anatomical structures between lianas and vines. Our study characterized three liana species (*Podranea ricasoliana*, *Cyclea hypoglauca*, and *Tetrastigma planicaule*) and three vine species (*Psychotria serpens*, *Passiflora caerulea*, and *Merremia hainanensis*) that inhabit the Limu Mountain Nature Reserve (Hainan, China), a representative tropical forest (Figure 1). For each plant type, the root tip diameters vary in a species-dependent manner, with root diameter of 111.3 ~ 207.9 μm and 106.5 ~ 183.3 μm between the thickest and the thinnest root species for vines and lianas, respectively (Figure 2). The root tip diameter and the anatomical traits, including cortex thickness, stele diameter, mean diameter of cortical cells, number of cortical cell layers, mean conduit diameter, number of conduits per stele, conduit density, and specific conductivity (*K*_s_) were investigated in each species. By analyzing the association between root tip diameter and these anatomical traits within and among species, the regulatory mechanism of the anatomical traits on the root tip diameter of lianas and vines were investigated and our hypotheses were further tested. Therefore, this study could have important theoretical implications for understanding the physiological and ecological functions of climbing plants in tropical forests.

## Materials and methods

### Study site

The research site was located in the Limushan Nature Reserve (109°39′–109°49′E, 19°07′–19°14′N), in Qiongzhong County, Hainan Province, China. The region has a tropical monsoon climate, with an average annual temperature of 23.1°C. The annual accumulated temperature is 8489.4°C, with the highest and lowest temperatures of 38.2°C and 1.5°C occurring in June and January, respectively (Wang et al., 2004). The rainy season occurs from May to October, with a precipitation of 1,809 mm, accounting for 83% of the annual precipitation. The region has an annual evaporation of 1,391–1,426 mm, a relative humidity of 87–88%, an annual sunshine duration of 1773.5–1918.3 h, and an annual average wind speed of 1.1 m/s (Qin, 2020). The study site was located mid-slope on a hill (100–200 m above sea level). The site soils are laterite and lateritic red, with a pH value of approximately 5, an organic matter ratio of 2.20%, and concentrations of total carbon, total nitrogen, total phosphorus, and total potassium of 1.28, 0.10, 0.08, and 2.24%, respectively (the soil data were provided by the Soil and Fertilizer Station, a section of the Department of Agriculture, Hainan Province, China).

### Root sampling

Our study characterized three woody liana species (*P. ricasoliana*, *C. hypoglauca*, and *T. planicaule*) and three herbaceous vine species (*P. serpens*, *P. caerulea*, and *M. hainanensis*) that grew at the study site. At the end of July 2019, for each species, three individual plants had a similar age or at a similar growth stage were randomly selected. The age (or growth stage) of the individual plant of vines and lianas was identified by the overall plant growth and stem diameter. In addition, for lianas, the same growth stage of individual plants could also be determined through the similar degree of lignification of stem and branches. The fine root segments of these individual plants were sampled as follows. First, the surface soil around the plants was gently removed by hand, and a spade or steel fork was used to pry the soil loose without damaging the roots of the study plants. Along with excavation of the main root system of each target plant, the surrounding soils were carefully loosened by hand to locate lateral root branches attached to the main roots. Complete fine root segments per plant from the 0 ~ 20 cm soil depths were randomly selected and carefully cut at the base using a pair of branch shears. After careful removal of soil particles, the fine root segments were immersed in formalin-aceto-alcohol (FAA) solution (90 ml of 50% ethanol, 5 ml of 100% glacial acetic acid, and 5 ml of 37% methanol) in reagent bottles, then placed on ice and immediately transported to the laboratory. The reagent bottles were stored at 4°C and the root anatomy was subsequently studied over a 3-month period.

### Measurement of root tip anatomical traits

In the laboratory, three complete fine root segments were randomly selected and removed from the FAA solution, then washed in deionized water to remove impurities. Root tips, identified as distal non-woody roots, were carefully dissected from the root samples using forceps, following the procedures described by Pregitzer et al. (2002) (Supplementary Figure S1). For each species, total 30–50 root tips from the three individual plants were collected and their surfaces were cleaned using a soft brush. A series of chemical processes, such as dehydration, dealcoholization, xylene removal, and embedding were applied to all root tip samples. Afterward, the root tips were embedded in paraffin, and slides with 8-μm-thick root sections were prepared using a microtome and then stained with safranin-fast green (2%; Gu et al., 2014a; Wang et al., 2018). Next, the slides were photographed under a compound microscope (BX-51; Olympus Corporation, Tokyo, Japan). Thirty intact cross sections, i.e., each from an individual root, were randomly selected as replications and for the measurement of functional traits. A range of functional traits, including root tip diameter, cortex thickness, stele diameter, mean diameter of cortical cells, number of layers of cortical cells, mean conduit diameter, and number of conduits per stele were measured using the Motic Images Advanced 3.2 software (Motic Corporation, Zhejiang, China). For each root tip, the mean value of each anatomical trait was obtained from measurements in three directions across the root cross-section. Based on the assumption that the stele is a perfect circle, the stele area was calculated using the circle area formula, taking the average stele diameter as the diameter of the circle. The conduit density was calculated as the ratio of the number of conduits per stele to the stele area (Kong et al., 2016). The specific conductivity (*K*_s_) was calculated using Hagen–Poiseuille’s law (Tyree and Ewers, 1991):


$$K_{\text{s}}=\left(\pi \rho /128\eta Aw\right)\sum_{i=1}^{n}\left(d_{i}^{4}\right)$$

where *K*_s_ is theoretical axial conductivity along a root tip, *ρ* is the density of water (where temperature was set at 18°C, consistent with the root respiration measurement), *η* is the dynamic viscosity, *d* is the diameter of the *i*th conduit, and *n* is the number of the conduits in the xylem.

### Data analysis

Thirty individual root tips, i.e., ten individual roots from each of the three individual plants, for each species were treated as the unit for data analysis in the current study. For each species, the average value and standard error of the root diameter and each anatomical trait were calculated from 30 replicates (i.e., 30 individual roots). One-way factorial analysis of variance (*p* = 0.05) was used to analyze the interspecific differences in functional traits of the root tips. Bivariate correlations among traits were evaluated using Pearson’s correlation coefficients (*r*) in the SPSS software (2010, V. 19.0, SPSS Inc., Cary, NC, United States), including the relationships (1) between root diameter versus both cortex thickness and stele diameter; (2) between cortex thickness versus both of mean cortical cell diameter and number of cortical cell layers; and (3) between stele diameter versus mean conduit diameter, number of conduits per stele, and conduit density. Additionally, these relationships were all examined by linear regressions and the adjusted regression coefficients (*R*^2^) were obtained. For each group of the intraspecific relationship, the data analyses were performed with 30 replicates for each species. The slopes of the regressions between root diameter and cortex thickness and stele diameter were compared *via* analysis of covariance (ANCOVA) in SPSS. Root tip functional traits were compared between vines and lianas using one-way factorial analysis of variance (*p* = 0.05) based on 90 replicates (i.e., 30 individual roots from each of the three species) for each life form. The interrelationships (*n* = 6, i.e., six species) between root tip diameter and multiple anatomical traits, such as cortex thickness, stele diameter, mean diameter of cortical cells, number of cortical cell layers, mean conduit diameter, and number of conduits per stele, were analyzed by principal component analysis (PCA) in the CANOCO software (CANOCO V. 4.5).

## Results

### Correlations of cortex thickness and stele diameter with root tip diameter

**Figure 3:**
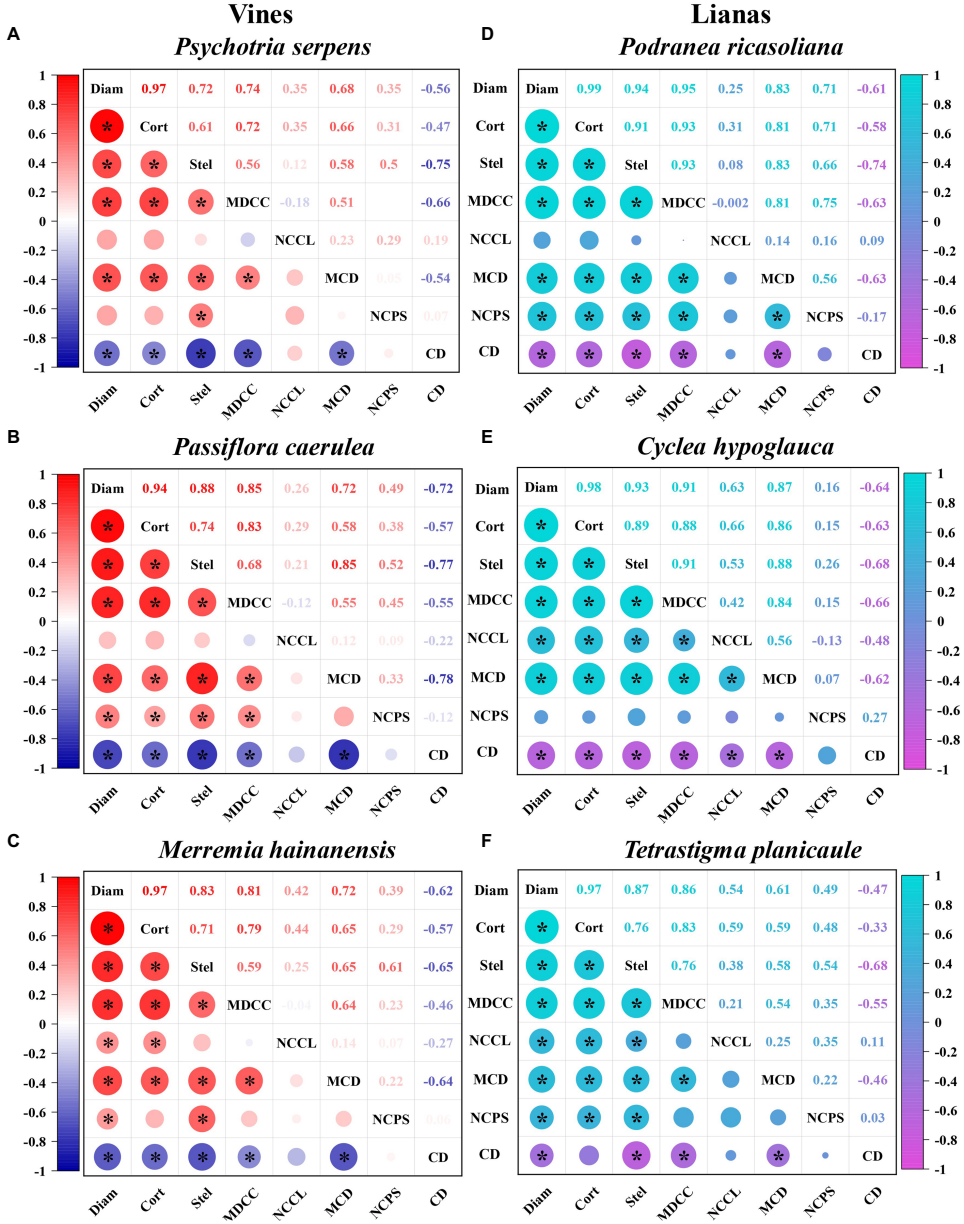
Intraspecific correlations of root tip diameter with cortex thickness, stele diameter, and cortical cells and conduit traits in three vine **(A-C)** (herbaceous climbing plants) and three liana **(D-F)** (woody climbing plants) species in tropical forest (*n* = 30). Color intensity represents the strength of the correlation, figures represent Pearson’s correlation coefficients (*r*), and significant correlations (*p* < 0.05) are indicated by “*”. See Supplementary Tables S2, S3 for adjusted regression coefficients (*R*^2^). Diam: root diameter; Cort: cortex thickness; Stel: stele diameter; MDCC: mean diameter of cortical cell; NCCL: number of cortical cell layer; MCD: mean conduit diameter; NCPS: number of conduits per stele; CD: conduit density.

The effects of cortex and stele morphology on root tip diameter can be analyzed by exploring the relationships between root tip diameter and cortex thickness and stele diameter. At the intra-species level, root tip cortex thickness and stele diameter both increased linearly with root diameter in six climbing plants (*r* = 0.94–0.99 and *r* = 0.72–0.94, respectively; *p* < 0.01, Figure 3). The slope for cortex thickness was much steeper than that for stele diameter (*p* < 0.01). The associations of root tip diameter with cortex thickness and with stele diameter were both strongest in *P. ricasoliana* (*r* = 0.99 and *r* = 0.94, respectively) among different species, but weakest in *P. caerulea* (*r* = 0.94, for the association with cortex thickness) and *P. serpens* (*r* = 0.72 for the association with stele diameter).

**Figure 4:**
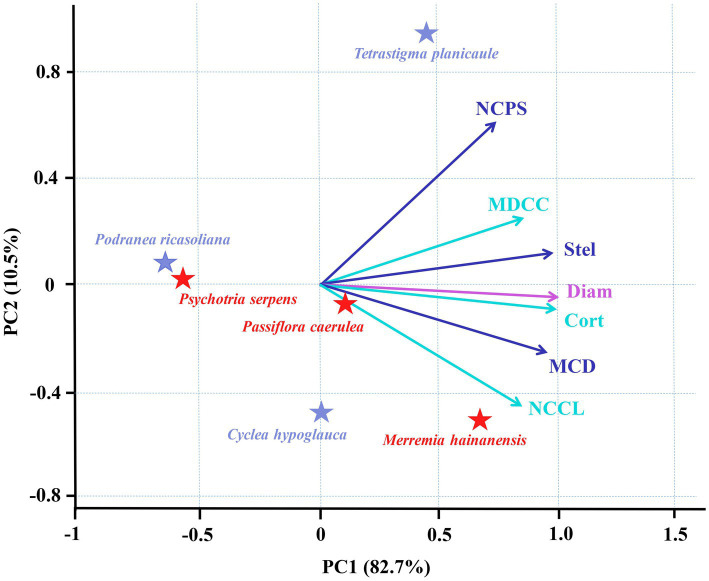
Principal component analyses (PCA) for root diameter and six anatomical traits of root tips in three vines (herbaceous climbing plants) and three lianas (woody climbing plants) in tropical forest (*n* = 6). All the abbreviations are the same as those described in Figure 3.

Among the three species for each vine and liana studied herein, there were increases in cortex thickness and stele diameter with root diameter (Figure 2). Such consistent trends among species resulted in the positive relationships between the root tip diameter and cortex thickness and stele diameter among the six climbing plants, with tighter associations between root tip diameter and cortex thickness (Figure 4). According to PCA, root diameter, cortex thickness, and stele diameter all contributed greatly to the first dimension, which could account for 82.7% of the total variation (Figure 4).

### Correlations of cortical cell traits and conduit traits with root tip diameter

The effects of cortical cell traits on cortex thickness and thus on root tip diameter were examined by exploring the relationships between cortical cell traits (i.e., mean diameter of cortical cells and number of cortical cell layers) and cortex thickness and root tip diameter. Our findings indicated that the mean diameter of cortical cells of root tips had significant positive correlations with cortex thickness (*r* = 0.72–0.93, *p* < 0.01) and with root tip diameter (*r* = 0.74–0.95, *p* < 0.01; Figure 3) within each species. In contrast, the intra-relationships between the number of cortical cell layers and cortex thickness and root tip diameter were relatively weaker, with significant effects observed only in the root tips of *C. hypoglauca* (*r* = 0.66 and *r* = 0.63, respectively), *T. planicaule* (*r* = 0.59 and *r* = 0.54, respectively), and *M. hainanensis* (*r* = 0.44 and *r* = 0.42, respectively).

The influence of conduit traits on stele diameter and thus on root tip diameter was assessed by exploring the relationships between conduit traits (i.e., mean conduit diameter, number of conduits per stele, and conduit density) and stele diameter and root tip diameter. The mean conduit diameter of the root tips showed positive correlations with stele diameter (*r* = 0.58–0.88, *p* < 0.05) and with root tip diameter (*r* = 0.61–0.87, *p* < 0.05) at the intraspecific level (Figure 3). However, the number of conduits per stele showed significant intra-associations with both stele diameter and root tip diameter only in *P. ricasoliana* (*r* = 0.66 and *r* = 0.71, respectively), *P. caerulea* (*r* = 0.52 and *r* = 0.49, respectively), *M. hainanensis* (*r* = 0.61 and *r* = 0.39, respectively), and *T. planicaule* (*r* = 0.54 and *r* = 0.49, respectively). In contrast, in *C. hypoglauca* and *P. serpens*, the number of conduits per stele was significantly correlated with the stele diameter at the intra-species level only in the latter species (*r* = 0.50) but was not correlated with root tip diameter in either species. Unlike the mean conduit diameter and the number of conduits per stele, conduit density was negatively correlated with both stele diameter (*r* = −0.65 to −0.77) and root tip diameter (*r* = −0.47 to −0.72) at the intraspecific level (Figure 3).

Based on the current limited number of observations, for each vine and liana, the interspecific variations of cortical cell and conduit traits are dependent on species and life forms. For example, the mean diameter of cortical cell and number of cortical cell layer was unchanged among three species of vines, but in lianas, it was more dependent on species (Supplementary Table S1). Among all six climbing plants examined herein, the root tip cortex thickness and root tip diameter were both positively correlated with the mean diameter of the cortical cells and the number of cortical cell layers (Figure 4). Consistent with the intraspecific associations, the mean diameter of cortical cells exhibited closer interspecific associations with cortex thickness and root tip diameter compared with the number of cortical cell layers. At the species level, stele diameter and root tip diameter were both positively correlated with the conduit diameter and the number of conduits per stele, with closer correlations with the conduit diameter (Figure 4). The PCA results demonstrated that the mean diameter of cortical cells and mean conduit diameter contributed greatly to the first dimension but their contribution was only slightly weaker than that of root tip diameter, cortex thickness, and stele diameter, all of which accounted for 82.7% of the total variation (Figure 4). In contrast, the number of cortical cell layers and number of conduits per stele contributed largely to the second dimension, which accounted for only 10.5% of the total variation. These results suggest that the first and second dimensions of the PCA were dominated by the diameter-and number-related traits, respectively, with diameter-related traits contributing substantially more to the total variation compared to the number-related traits.

### Difference between vines and lianas

**Figure 5:**
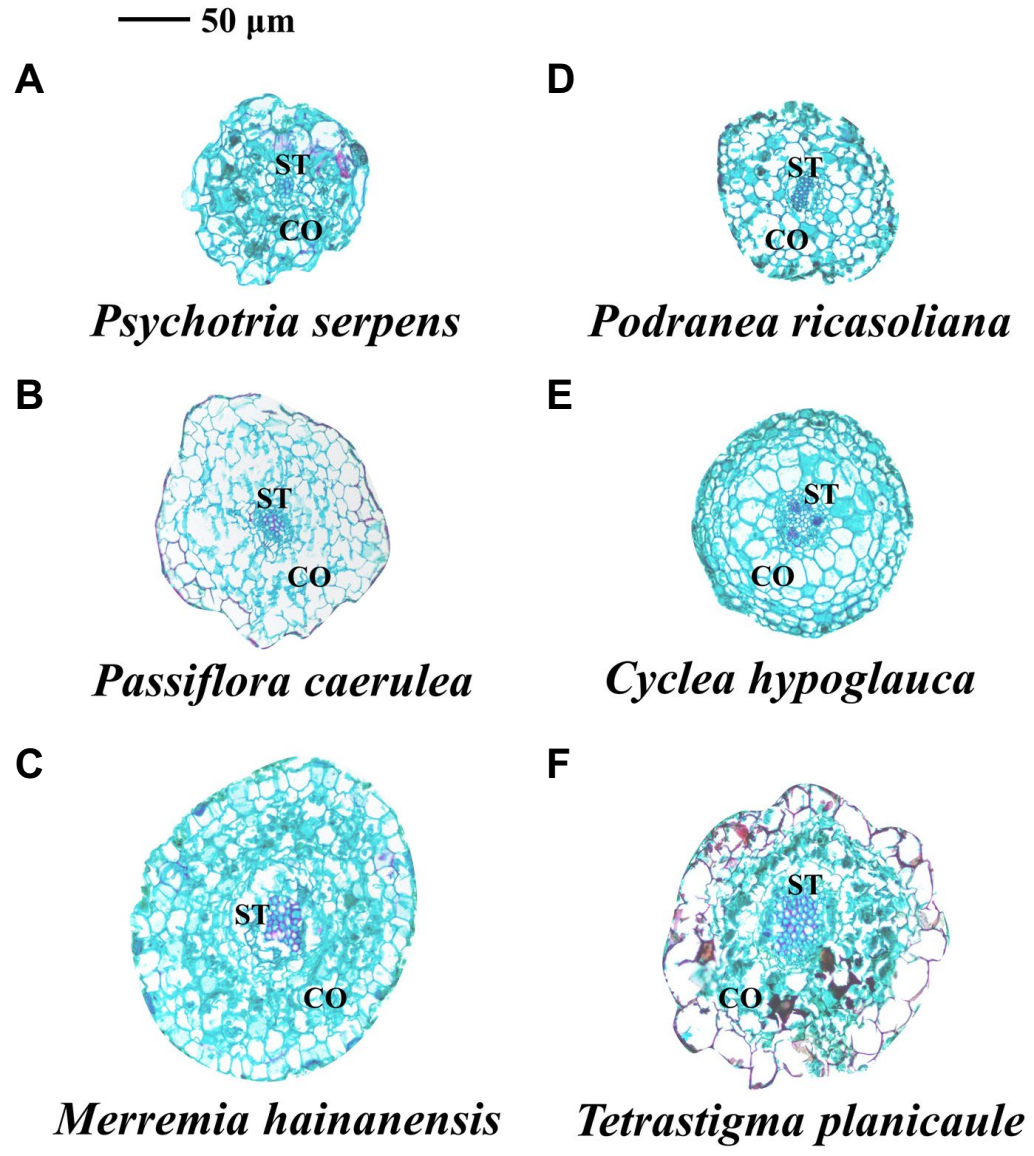
Anatomical structure of root tips in three vine **(A–C)** (herbaceous climbing plants) and three liana **(D–F)** (woody climbing plants) species in tropical forest. CO: cortex; ST: stele.

For vines and lianas, the root tip diameter, cortex thickness, and stele diameter exhibited similar trends among species. The root tip diameter values exhibited the following ascending order in vine species: *P. serpens* < *P. caerulea* < *M. hainanensis*. The root tip diameter of lianas had the following order: *P. ricasoliana* < *C. hypoglauca* < *T. planicaule* (Figures 2, 5). Based on the small sample size in the current study, the differences in root tip diameter, cortex thickness, and stele diameter were not significant between these two plant types (Figure 2). But the ratio of bilateral cortex thickness to root tip diameter (3.6% higher) and conduit diameter (12.0% higher) in vines were significantly higher than in lianas (Table 1). However, lianas exhibited 9.6% higher stele diameter to root tip diameter ratios, 22.2% greater conduit numbers, and 23.8% higher conduit density compared with vines, which were all significantly different between life forms (*p* < 0.05). In contrast, no significant difference was observed for the *K*_s_ between lianas and vines (Table 1). Note that the replicates are from only three individual plants per species limited these comparisons between life forms. Furthermore, the correlations between these functional traits also varied considerably between the two plant types. Specifically, the correlation coefficients between root tip diameter and cortex thickness and stele diameter, and between cortex thickness and mean diameter of the cortical cells were generally higher in lianas (*r* = 0.87–0.99) than in vines (*r* = 0.72–0.97, Figure 3).

**Table 1:** Mean values and coefficient of variations of cortex and stele proportions, and cortical cell and conduit traits of root tips in vine (herbaceous climbing plants) and liana (woody climbing plants) in tropical forest (*n* = 90).

Anatomical traits	Vines		Lianas	
	Mean	CV%	Mean	CV%
Cortex proportion and cortical cell traits				
Ratio of bilateral cortex thickness to root tip diameter (%)	76.94a	1.97	74.27b	1.51
Mean diameter of cortical cell (μm)	10.58a	9.83	9.73a	24.29
Number of cortical cell layer	5.88a	15.93	5.83a	8.3
Stele proportion and conduit traits				
Ratio of stele diameter to root tip diameter (%)	22.44b	11.18	24.81a	10.42
Mean conduit diameter (μm)	4.61a	17.7	4.12b	16.64
Number of conduits per stele	15.29b	37.45	19.66a	45.58
Conduit density (no. mm^−2^)	0.02b	39.03	0.03a	33.88
*K*s (Kg m^−1^ Mpa^−1^ s^−1^)	0.17a	27.11	0.15a	40.14

Significant differences (*p* < 0.05) between vines and lianas are indicated by different lower-case letters.

## Discussion

### Cortex thickness has a stronger influence on the changes in root tip diameter compared to stele diameter

Our findings indicated that the root tip cortex thickness and stele diameter of the six climbing plants were significantly positively correlated with root tip diameter within and among species, with closer relationships observed between cortex thickness and root tip diameter (Figures 3, 4; Supplementary Table S2). These results confirmed the first hypothesis that cortex have stronger effects than stele in the regulation of root diameter in climbing plants. However, for climbing plants, studies on the linkage of root tip diameter with anatomical traits are extremely lacking. But Wang et al. (2020) studied three temperate climbing species and found that cortex thickness of root tips had more similar interspecific variations to root tip diameter than stele diameter. This may indirectly support our findings. Closer inter-associations between root tip diameter and cortex thickness have also been reported in trees, for example, among 27 tropical tree species and 96 subtropical tree species (Kong et al., 2014; Gu et al., 2014a). However, regarding the intraspecific relationships, the relationship strength in climbing plants was different from those of the temperate tree species examined by Wang et al. (2018). Specifically, the authors found that the correlation strengths of root diameter with cortex thickness and stele diameter were more dependent on species. It is not clear whether such a disagreement is caused by climate factors or the inherent differences between plant types (i.e., trees and climbing plants). However, our study consistently identified a stronger influence of cortex thickness on root tip diameter in both lianas and vines, suggesting that the root tips of climbing plants preferentially invest in a thicker cortex rather than a thicker stele during root development. Species with a thick cortex generally possess better water and nutrient acquisition capacities because a thick cortex provides ample space for mycorrhizal colonization (Comas et al., 2012; Zhou et al., 2021). Therefore, we speculate that the strong influence of cortex thickness on root tip diameter in climbing plants enhances the resource acquisition capacities of individual plants.

There are mainly three reasons for the stronger relationship between cortex thickness and root tip diameter at the inter- and intra-specific levels in climbing plants. First, the greater proportion of cortex in the cross-sectional area of root tips, as found in the current and previous studies (Hummel et al., 2007; Gu et al., 2014a; Wang et al., 2020), could directly explain their closer linkage with root tip diameter. Second, the cortex is wrapped around the stele and therefore is more susceptible to environmental stress (Kondo et al., 2000; Enstone et al., 2003; Lux et al., 2004; Gu et al., 2014a). The high sensitivity and susceptibility of the cortex to environmental changes caused by its lateral location may explain its closer correlation with root tip diameter in climbing plants. Previous studies on tropical species also confirmed that the change in root diameter caused by water deficiencies was more consistent with changes in cortex thickness compared to stele diameter in *Astragalus gombiformis* and *Stipa lagascae* (Boughalleb et al., 2014, 2015). Third, the strength of the relationship between root tip diameter and cortex thickness in climbing plants may be related to their unique root physiological regulation strategy. Specifically, changes in root cortex thickness are typically accompanied by multifaceted physiological changes in roots, such as water and nutrient uptake, lateral transport resistance, and energy expenditure (Lascaris and Deacon, 1991; Elliott et al., 1993; Huang and Eissenstat, 2000; Kong et al., 2017; Schneider et al., 2017). Therefore, the stronger linkage between cortex thickness and root tip diameter in climbing plant root tips could be an effective strategy to modulate plant growth and physiology. However, the individual root samples were taken from only three individual plants per species growing in the similar locations, and therefore the variation of root tip functional traits observed in the current study may describe a lot of intra-individual variation. But the variation of the environment could have been greater for some of these species than others. The generality of these results and whether they apply in other species of climbing plants will require more extensive investigations with large sample numbers.

### Diameter-related traits of cortical cells and conduits have a stronger influence on root tip diameter than number-related traits

The results of this study confirmed the second hypothesis that the diameter-related traits of cortical cells and conduits play more important roles in the regulation of cortex thickness and stele diameter, respectively, than the number-related traits, and therefore contribute greatly to the variation of root tip diameter in climbing plants (Figures 3, 4; Supplementary Tables S2, S3). There has been a lack of studies on the associations of anatomical traits of root tips in climbing plants. But previous studies on self-supporting plants have reported that the change in cortical cell diameter represents a lower energy expenditure compared to the number of cortical cell layers, because the former and latter are attributable to variations in vacuole size and the cytoplasm, respectively (Chimungu et al., 2014). Cortical cell diameter was also found to respond more sensitively than the number of cortical cell layers when the roots of *Zea mays* (Chimungu et al., 2014; Saengwilai et al., 2014), *Malus hupehensi*, and *Malus xiaojinensis* (Wang, 2012) experienced environmental stresses such as water, nitrogen, and zinc deficiencies. Therefore, the closer relationship between cortical cell diameter and root tip diameter observed in our study indicated that the root tip of climbing plants plays a crucial role in regulating growth and metabolism in a cost-efficient manner. When comparing the diameter and number of conduits, the former plays a more important role in determining stele diameter and then influencing root tip diameter. Based on Hagen–Poiseuille’s law, the enlargement of conduit diameter can efficiently improve root transport (Tyree and Ewers, 1991). Furthermore, it appears that climbing plant root tips could enlarge their size to efficiently improve their transport ability with increasing root tip diameter. Climbing plants are thought to be more vulnerable to environmental stresses (e.g., drought) than other plant forms (Ewers, 1985; Ewers et al., 1990; Jiménez-Castillo et al., 2006; Hu et al., 2010), due to their extremely wide vessels, which result in higher risks of embolisms than small vessels in self-supporting plants (Ewers et al., 1990; Hacke et al., 2000). The close linkage between conduit diameter and root tip diameter in our study further illustrates that the vulnerabilities of climbing plants to environmental stresses are likely severer in thick-root species and individuals with well-developed root tips. This, however, requires further investigation due to the small number of observations in the current study.

In addition, we noted that the root tip stele diameter was negatively associated with conduit density in climbing plants, which was the opposite to those with conduit diameter and number (Figures 3, 4; Supplementary Table S3). These findings are in agreement with previous studies conducted on subtropical tree species (Kong et al., 2014). Species with large conduit density and small conduits are considered to have weak transport but larger long-term water use efficiency and better adaptability to arid conditions (Poorter et al., 2010; Kong et al., 2014; Pfautsch et al., 2016a,b; Zhou et al., 2021). In our study, the correlations between conduit traits and root tip diameter were similar to those with stele diameter, suggesting that the species of climbing plants with thinner root tips would have more advantages in water and nutrient uptake compared to vertical transport, which is consistent with previous studies on self-supporting plants (Roumet et al., 2016). These findings suggest that conduit traits are likely responsible for the trade-offs of absorption and transport in the root tips of climbing plants.

### Higher stele proportion and closer relationships between functional traits in lianas (woody climbing plants) than vines (herbaceous climbing plants)

In the present study, root tips of lianas have smaller but denser conduits in a thicker stele when compared to vines with a similar root tip diameter, and the associations of root diameter with anatomical traits were stronger in lianas than in vines (Figure 3; Table 1; Supplementary Table S2), supported our third hypothesis. These features of liana root tips may be an important adaptation of anatomical structure in response to climatic stress. Lianas are more sensitive to water and temperature stresses induced by climate factors than vines due to their longer life cycle and lower belowground storage capacities (Jiménez-Castillo et al., 2006; Hu et al., 2010; Angyalossy et al., 2012). Therefore, these closer relationships between root tip diameter and anatomical traits in the root tips of lianas appear to be an effective strategy to better cope with climate-induced environmental stressors such as drought and cold stress. The wide vessels of climbing plants are typically vulnerable to drought-and cold-induced embolism (Hu et al., 2010). Our findings indicated that lianas had significantly smaller but denser conduits in a thicker stele compared to vines, which may reduce the damage caused by climatic stress (e.g., drought and cold conditions). Surprisingly, no significant difference was observed for the *K*_s_ between lianas and vines at the similar root tip diameters. Therefore, for similar diameter of root tips of lianas and vines, the difference in xylem observed between the two plant types suggests that these climbing plants possess diverse vertical transport strategies but similar transport abilities.

Compared with climatic changes, Bhattarai and Vetaas (2003) reported that herbaceous species, including vines, may be more sensitive to local environmental stresses such as nutrient and water deficiencies in soil. For self-supporting species, woody plants can grow deeper in soil to search for water under local drought conditions, whereas herbaceous plants remain in shallow soils and therefore fully experience the adverse effects of drought conditions (Ansley et al., 2014). In climbing plants, the root tips of vines possess significantly higher ratios of bilateral cortex thickness to root diameter when compared to lianas with similar root tip diameters (Table 1). Therefore, we inferred that vine root tips tend to develop a thicker cortex during growth in order to cope with local environmental stress, thus providing ample space for both the temporary storage of water and minerals and mycorrhizal colonization (Comas et al., 2012). Similarly, for self-supporting species, root N uptake rate has been reported to be higher in herbaceous plants than in woody plants (Ma et al., 2018). Therefore, vines and lianas possess distinct growth strategies, which was reflected in the differences in root tip anatomical cross-sectional structure, as well as in the correlations of these anatomical traits with root tip diameter. Taken together, our findings provide important insights into the influence of different anatomical structures on root tip size in lianas and vines in tropical ecosystems. This study is a detailed investigation of anatomical trait relationships of a limited number of study plants, but more work is needed to understand the extent of these relationships and patterns across environmental gradients and among other climbing plants.

## Data availability statement

The raw data supporting the conclusions of this article will be made available by the authors, without undue reservation.

## Author contributions

WW and SW conceived and designed this research project. HX, WW, and LT performed the data analysis. WW, SW, YW, and ZL completed the field work. HX and SW performed the laboratory experiment and have contributed equally to this work and share first authorship. WW and LT supervised this work. HX, SW, LT, YW, ZL, and WW contributed to the revisions and comments concerning the manuscript. All authors contributed to the article and approved the submitted version.

## Funding

This research was funded by the National Natural Science Foundation of China (31901301), Hainan Provincial Natural Science Foundation of China for High-level Talents (2019RC159), and Scientific Research Foundation for Hainan University [KYQD (ZR)1987].

## Conflict of interest

The authors declare that the research was conducted in the absence of any commercial or financial relationships that could be construed as a potential conflict of interest.

## Publisher’s note

All claims expressed in this article are solely those of the authors and do not necessarily represent those of their affiliated organizations, or those of the publisher, the editors and the reviewers. Any product that may be evaluated in this article, or claim that may be made by its manufacturer, is not guaranteed or endorsed by the publisher.

## References

[ref1] Angyalossy V. Angeles G. Pace M. R. Lima A. C. Dias-Leme C. L. Lohmann L. G.. (2012). An overview of the anatomy, development and evolution of the vascular system of lianas. Plant Ecol. Divers. 5, 167–182. doi: 10.1080/17550874.2011.615574

[ref2] Ansley R. J. Boutton T. W. Jacoby P. W. (2014). Root biomass and distribution patterns in a semi-arid mesquite savanna: responses to long-term rainfall manipulation. Rangel. Ecol. Manag. 67, 206–218. doi: 10.2111/REM-D-13-00119.1

[ref3] Bhattarai K. R. Vetaas O. R. (2003). Variation in plant species richness of different life forms along a subtropical elevation gradient in the Himalayas, East Nepal. Glob. Ecol. Biogeogr. 12, 327–340. doi: 10.1046/j.1466-822X.2003.00044.x

[ref4] Boughalleb F. Abdellaoui R. Ben-Brahim N. Neffati M. (2014). Anatomical adaptations of *Astragalus gombiformis* Pomel under drought stress. Open Life Sci. 9, 1215–1225. doi: 10.2478/s11535-014-0353-7

[ref5] Boughalleb F. Abdellaoui R. Hadded Z. Neffati M. (2015). Anatomical adaptations of the desert species *Stipa lagascae* against drought stress. Biologia 70, 1042–1052. doi: 10.1515/biolog-2015-0125

[ref6] Bowsher A. W. Miller B. J. Donovan L. A. (2016). Evolutionary divergences in root system morphology, allocation, and nitrogen uptake in species from high-versus low-fertility soils. Funct. Plant Biol. 43, 129–140. doi: 10.1071/FP15162, PMID: 32480447

[ref7] Burton A. J. Jarvey J. C. Jarvi M. P. Zak D. R. Pregitzer K. S. (2012). Chronic N deposition alters root respiration-tissue N relationship in northern hardwood forests. Glob. Chang. Biol. 18, 258–266. doi: 10.1111/j.1365-2486.2011.02527.x

[ref8] Chimungu J. G. Brown K. M. Lynch J. P. (2014). Large root cortical cell size improves drought tolerance in maize. Plant Physiol. 166, 2166–2178. doi: 10.1104/pp.114.250449, PMID: 25293960 PMC4256844

[ref9] Collins C. G. Wright S. J. Wurzburger N. (2016). Root and leaf traits reflect distinct resource acquisition strategies in tropical lianas and trees. Oecologia 180, 1037–1047. doi: 10.1007/s00442-015-3410-7, PMID: 26254258

[ref10] Comas L. H. Mueller K. E. Taylor L. L. Midford P. E. Callahan H. S. Beerling D. J. (2012). Evolutionary patterns and biogeochemical significance of angiosperm root traits. Int. J. Plant Sci. 173, 584–595. doi: 10.1086/665823

[ref11] Dong X. Y. Wang H. F. Gu J. C. Wang Y. Wang Z. Q. (2015). Root morphology, histology and chemistry of nine fern species (Pteridophyta) in a temperate forest. Plant Soil 393, 215–227. doi: 10.1007/s11104-015-2484-7

[ref12] Elliott G. A. Robson A. D. Abbott L. K. (1993). Effects of phosphate and nitrogen application on death of the root cortex in spring wheat. New Phytol. 123, 375–382. doi: 10.1111/j.1469-8137.1993.tb03748.x

[ref13] Enstone D. E. Peterson C. A. Ma F. (2003). Root endodermis and exodermis: structure, function, and responses to the environment. J. Plant Growth Regul. 21, 335–351. doi: 10.1007/s00344-003-0002-2

[ref14] Esau K. (1977). Anatomy of Seed Plants. 2nd Edn. New York, NY: Wiley, 215–255.

[ref15] Ewers F. W. (1985). Xylem structure and water conduction in conifer trees, dicot trees, and lianas. IAWA Bulletin 6, 309–317.

[ref16] Ewers F. W. Fisher J. B. Chiu S. T. (1990). A survey of vessel dimensions in stems of tropical lianas and other growth forms. Oecologia 84, 544–552. doi: 10.1007/BF00328172, PMID: 28312972

[ref17] Genre A. Chabaud M. Faccio A. Barker D. G. Bonfante P. (2008). Prepenetration apparatus assembly precedes and predicts the colonization patterns of arbuscular mycorrhizal fungi within the root cortex of both *Medicago truncatula* and *Daucus carota*. Plant Cell 20, 1407–1420. doi: 10.1105/tpc.108.059014, PMID: 18515499 PMC2438458

[ref19] Gu J. C. Xu Y. Dong X. Y. Wang H. F. Wang Z. Q. (2014a). Root diameter variations explained by anatomy and phylogeny of 50 tropical and temperate tree species. Tree Physiol. 34, 415–425. doi: 10.1093/treephys/tpu019, PMID: 24695727

[ref20] Guo D. L. Xia M. X. Wei X. Chang W. J. Liu Y. Wang Z. Q. (2008b). Anatomical traits associated with absorption and mycorrhizal colonization are linked to root branch order in twenty-three Chinese temperate tree species. New Phytol. 180, 673–683. doi: 10.1111/j.1469-8137.2008.02573.x, PMID: 18657210

[ref21] Hacke U. G. Sperry J. S. Pittermann J. (2000). Drought experience and cavitation resistance in six shrubs from the Great Basin, Utah. Basic Appl. Ecol. 1, 31–41. doi: 10.1078/1439-1791-00006

[ref22] Hu L. Li M. G. Li Z. (2010). Geographical and environmental gradients of lianas and vines in China. Glob. Ecol. Biogeogr. 19, 554–561. doi: 10.1111/j.1466-8238.2010.00527.x

[ref23] Huang B. Eissenstat D. M. (2000). Linking hydraulic conductivity to anatomy in plants that vary in specific root length. J. Am. Soc. Hortic. Sci. 125, 260–264. doi: 10.21273/JASHS.125.2.260

[ref24] Hummel I. Vile D. Violle C. Devaux J. Ricci B. Blanchard A.. (2007). Relating root structure and anatomy to whole-plant functioning in 14 herbaceous Mediterranean species. New Phytol. 173, 313–321. doi: 10.1111/j.1469-8137.2006.01912.x, PMID: 17204078

[ref25] Ingwell L. L. Joseph Wright S. Becklund K. K. Hubbell S. P. Schnitzer S. A. (2010). The impact of lianas on 10 years of tree growth and mortality on Barro Colorado Island, Panama. J. Ecol. 98, 879–887. doi: 10.1111/j.1365-2745.2010.01676.x

[ref26] Jia S. X. Wang Z. Q. Li X. P. Sun Y. Zhang X. Liang A. (2010). N fertilization affects on soil respiration, microbial biomass and root respiration in *Larix gmelinii* and *Fraxinus mandshurica* plantations in China. Plant Soil 333, 325–336. doi: 10.1007/s11104-010-0348-8

[ref27] Jiménez-Castillo M. Wiser S. K. Lusk C. H. (2006). Elevational parallels of latitudinal variation in the proportion of lianas in woody floras: Elevational variation in floristic importance of lianas. J. Biogeogr. 34, 163–168. doi: 10.1111/j.1365-2699.2006.01570.x

[ref28] Kondo M. Aguilar A. Abe J. Morita S. (2000). Anatomy of nodal roots in tropical upland and lowland rice varieties. Plant Prod. Sci. 3, 437–445. doi: 10.1626/pps.3.437

[ref29] Kong D. L. Ma C. G. Zhang Q. Li L. Chen X. Y. Zeng H.. (2014). Leading dimensions in absorptive root trait variation across 96 subtropical forest species. New Phytol. 203, 863–872. doi: 10.1111/nph.12842, PMID: 24824672

[ref30] Kong D. L. Wang J. J. Kardol P. Wu H. F. Zeng H. Deng X. B.. (2016). Economic strategies of plant absorptive roots vary with root diameter. Biogeosciences 13, 415–424. doi: 10.5194/bg-13-415-2016

[ref31] Kong D. L. Wang J. J. Zeng H. Liu M. Z. Miao Y. Wu H. F.. (2017). The nutrient absorption–transportation hypothesis: optimizing structural traits in absorptive roots. New Phytol. 213, 1569–1572. doi: 10.1111/nph.14344, PMID: 27859373

[ref32] Lascaris D. Deacon J. W. (1991). Relationship between root cortical senescence and growth of wheat as influenced by mineral nutrition, *Idriella bolleyi* (Sprague) von Arx and pruning of leaves. New Phytol. 118, 391–396. doi: 10.1111/j.1469-8137.1991.tb00020.x

[ref33] Lux A. Luxova M. Abe J. Morita S. (2004). Root cortex: structural and functional variability and responses to environmental stress. Root Res. 13, 117–131. doi: 10.3117/rootres.13.117

[ref34] Ma Z. Q. Guo D. L. Xu X. L. Lu M. Z. Bardgett R. D. Eissenstat D. M.. (2018). Evolutionary history resolves global organization of root functional traits. Nature 555, 94–97. doi: 10.1038/nature25783, PMID: 29466331

[ref35] Pfautsch S. Harbusch M. Wesolowski A. Smith R. Macfarlane C. Tjoelker M. G.. (2016a). Climate determines vascular traits in the ecologically diverse genus *eucalyptus*. Ecol. Lett. 19, 240–248. doi: 10.1111/ele.12559, PMID: 26743135

[ref36] Pfautsch S. Macfarlane C. Harbusch M. Wesolowski A. Smith R. Boer M.. (2016b). Vessel diameter and related hydraulic traits of 31 *eucalyptus* species arrayed along a gradient of water availability. Ecology 97:1626. doi: 10.1890/16-0147.1, PMID: 27859219

[ref37] Phillips O. L. Martinez R. V. Arroyo L. Baker T. R. Killeen T. Lewis S. L.. (2002). Increasing dominance of large lianas in Amazonian forests. Nature 418, 770–774. doi: 10.1038/nature00926, PMID: 12181565

[ref38] Poorter L. McDonald I. Alarcón A. Fichtler E. Licona J. Peña-Claros M.. (2010). The importance of wood traits and hydraulic conductance for the performance and life history strategies of 42 rainforest tree species. New Phytol. 185, 481–492. doi: 10.1111/j.1469-8137.2009.03092.x, PMID: 19925555

[ref39] Pregitzer K. S. Deforest J. L. Burton A. J. Allen M. F. Ruess R. W. Hendrick R. L. (2002). Fine root architecture of nine north American trees. Ecol. Monogr. 72, 293–309. doi: 10.1890/0012-9615(2002)072[0293:FRAONN]2.0.CO;2

[ref40] Putz F. E. (1983). Liana biomass and leaf area of a “tierra firme” forest in the Rio Negro basin, Venezuela. Biotropica 15, 185–189. doi: 10.2307/2387827

[ref41] Putz F. E. (1984). The natural history of lianas on Barro Colorado Island, Panama. Ecology 65, 1713–1724. doi: 10.2307/1937767

[ref42] Qin X. T.. (2020). Flora Analysis of Bryophytes in Limushan Nature Reserve. Haikou: Hainan University Press.

[ref43] Roumet C. Birouste M. Picon-Cochard C. Ghestem M. Osman N. Vrignon Brenas S.. (2016). Root structure–function relationships in 74 species: evidence of a root economics spectrum related to carbon economy. New Phytol. 210, 815–826. doi: 10.1111/nph.13828, PMID: 26765311

[ref44] Saengwilai P. Nord E. A. Chimungu J. G. Brown K. M. Lynch J. P. (2014). Root cortical aerenchyma enhances nitrogen acquisition from low-nitrogen soils in maize. Plant Physiol. 166, 726–735. doi: 10.1104/pp.114.241711, PMID: 24891611 PMC4213101

[ref45] Schneider H. M. Wojciechowski T. Postma J. A. Brown K. M. Lücke A. Zeisler V.. (2017). Root cortical senescence decreases root respiration, nutrient content and radial water and nutrient transport in barley: cortical senescence reduces respiration, nutrient content and radial transport. Plant Cell Environ. 40, 1392–1408. doi: 10.1111/pce.12933, PMID: 28164319

[ref46] Schnitzer S. A. Bongers F. (2002). The ecology of lianas and their role in forests. Trends Ecol. Evol. 17, 223–230. doi: 10.1016/S0169-5347(02)02491-6

[ref47] Schnitzer S. A. Bongers F. (2011). Increasing liana abundance and biomass in tropical forests: emerging patterns and putative mechanisms: increasing lianas in tropical forests. Ecol. Lett. 14, 397–406. doi: 10.1111/j.1461-0248.2011.01590.x, PMID: 21314879

[ref48] Schnitzer S. A. Kuzee M. E. Bongers F. (2005). Disentangling above-and below-ground competition between lianas and trees in a tropical forest. J. Ecol. 93, 1115–1125. doi: 10.1111/j.1365-2745.2005.01056.x

[ref49] Smith-Martin C. M. Bastos C. L. Lopez O. R. Powers J. S. Schnitzer S. A. (2019). Effects of dry-season irrigation on leaf physiology and biomass allocation in tropical lianas and trees. Ecology 100:e02827. doi: 10.1002/ecy.2827 31325383

[ref50] Tyree M. T. Ewers F. W. (1991). The hydraulic architecture of trees and other woody plants. New Phytol. 119, 345–360. doi: 10.1111/j.1469-8137.1991.tb00035.x

[ref51] Wang J. H.. (2012). The Morphological and Physiological Responses of Apple Rootstocks to Zinc-Deficiency Stress and Regulation of IAA in the Root Growth. Taian: Shandong Agriculture University Press.

[ref52] Wang W. N. Wang Y. Hoch G. Wang Z. Q. Gu J. C. (2018). Linkage of root morphology to anatomy with increasing nitrogen availability in six temperate tree species. Plant Soil 425, 189–200. doi: 10.1007/s11104-018-3563-3

[ref300] Wang L. J. Hong M. L. Wang J. C. Lv S. Q. Shi H. T. (2004). Diversity and Fauna Analysis of Amphibians in Limushan Nature Reserve of Hainan Province. Chinese J. Zool. 39, 54–57., PMID: 27149034

[ref53] Wang Y. M. Wang Y. Wang S. Y. Gao G. Q. Gu J. C. (2020). Fine root anatomical and morphological traits of three temperate liana species in northeastern China. J. Beijing For. Univ. 42, 42–49. doi: 10.12171/j.1000-1522.20190419

[ref54] Wright S. J. Calderón O. Hernandéz A. Paton S. (2004). Are lianas increasing in importance in tropical forests? A 17-year record from Panama. Ecology 85, 484–489. doi: 10.1890/02-0757

[ref55] Zhou M. Bai W. Li Q. Guo Y. Zhang W. H. (2021). Root anatomical traits determined leaf-level physiology and responses to precipitation change of herbaceous species in a temperate steppe. New Phytol. 229, 1481–1491. doi: 10.1111/nph.16797, PMID: 32645210

