# Supplementary material for: Differential influence of cortex and stele components on root tip diameter in different types of tropical climbing plants
Source: Front Plant Sci. 2022 Aug 31;13:961214. doi: 10.3389/fpls.2022.961214 (PMC9470880; doi:10.3389/fpls.2022.961214)
Supplement: Supplementary file 1 [file Data_Sheet_1.docx]

**Supplementary Material**





**Figure S1** Root samples of fine root segments of three vine (A-C) (herbaceous climbing plants) and three liana (D-F) (woody climbing plants) species in tropical forest. Root tips are indicated by arrows.

|  | **Vines** | | |  | **Lianas** | | |
| --- | --- | --- | --- | --- | --- | --- | --- |
|  | *Psychotria serpens* | *Passiflora caerulea* | *Merremia hainanensis* |  | *Podranea ricasoliana* | *Cyclea hypoglauca* | *Tetrastigma planicaule* |
| **Cortical cell traits** |  |  |  |  |  |  |  |
| Mean diameter of cortical cell (μm) | 9.38 ± 0.44 a | 11.27 ± 0.55 a | 11.08 ± 0.63 a |  | 7.29 ± 0.57 b | 9.88 ± 0.50 a | 12.02 ± 0.67 a |
| Number of cortical cell layer | 4.90 ± 0.14 a | 5.97 ± 0.15 a | 6.77 ± 0.19 a |  | 5.37 ± 0.20 a | 6.33 ± 0.17 a | 5.80 ± 0.21 b |
| **Conduit traits** |  |  |  |  |  |  |  |
| Mean conduit diameter (μm) | 3.88 ± 0.11 a | 4.46 ± 0.14 a | 5.49 ± 0.17 a |  | 3.33 ± 0.12 b | 4.44 ± 0.22 a | 4.59 ± 0.21 b |
| Number of conduits per stele | 9.07 ± 0.65 b | 16.47 ± 1.04 a | 20.33 ± 1.81 b |  | 14.47 ± 0.81 a | 14.50 ± 1.14 a | 30.00 ± 2.01 a |
| Conduit density (no. mm^-2^) | 0.025 ± 0.003 b | 0.023 ± 0.003 a | 0.011 ± 0.001 b |  | 0.035 ± 0.003 a | 0.020 ± 0.003 a | 0.021 ± 0.003 a |
| *K*s (Kg m^-1^ Mpa-1 s^-1^) | 0.12 ± 0.01 a | 0.17 ± 0.01 a | 0.21 ± 0.02 a |  | 0.09 ± 0.01 a | 0.15 ± 0.02 a | 0.22 ± 0.05 a |

**Table S1** Mean values of cortical cell traits and conduit traits of root tips in three vine (herbaceous climbing plants) and three liana (woody climbing plants) species in tropical forest (*n* = 30).

Significant differences (*p* <0.05) between treatments are indicated by different lower-case letters.

**Table S2** Adjusted regression coefficients (*R*^2^) between root tip diameter and anatomical traits in three vine (herbaceous climbing plants) and three liana (woody climbing plants) species in tropical forest (*n* = 30).

|  |  |  |  | Root diameter |  |  |  |
| --- | --- | --- | --- | --- | --- | --- | --- |
|  | Cortex thickness | Stele  diameter | Mean diameter of cortical cell | Number of cortical cell layer | Mean conduit diameter | Number of conduits per stele | Conduit  density |
| **Vines** |  |  |  |  |  |  |  |
| *Psychotria serpens* | 0.94 ** | 0.49 ** | 0.53 ** | 0.09 | 0.44 ** | 0.09 | 0.30 ** |
| *Passiflora caerulea* | 0.89 ** | 0.77 ** | 0.72 ** | 0.03 | 0.51 ** | 0.21 ** | 0.43 ** |
| *Merremia hainanensis* | 0.94 ** | 0.67 ** | 0.63 ** | 0.15 * | 0.50 ** | 0.12 * | 0.13 * |
| **Lianas** |  |  |  |  |  |  |  |
| *Podranea ricasoliana* | 0.99 ** | 0.88 ** | 0.90 ** | 0.03 | 0.67 ** | 0.49 ** | 0.34 ** |
| *Cyclea hypoglauca* | 0.96 ** | 0.87 ** | 0.81 ** | 0.38 ** | 0.75 ** | -0.01 | 0.42 ** |
| *Tetrastigma planicaule* | 0.94 ** | 0.75 ** | 0.73 ** | 0.27 ** | 0.34 ** | 0.21 ** | 0.15 * |

*: *p* <0.05, **: *p* <0.01

**Table S3** Adjusted regression coefficients (*R*^2^) between root tip cortex thickness and cortical cell traits, and between stele diameter and conduit traits in three vine (herbaceous climbing plants) and three liana (woody climbing plants) species in tropical forest (*n* = 30).

|  | Cortex thickness | |  | Stele diameter | | |
| --- | --- | --- | --- | --- | --- | --- |
|  | Mean diameter of cortical cell | Number of cortical cell layer |  | Mean conduit diameter | Number of conduits per stele | Conduit density |
| **Vines** |  |  |  |  |  |  |
| *Psychotria serpens* | 0.50 ** | 0.09 |  | 0.33 ** | 0.23 ** | 0.53 ** |
| *Passiflora caerulea* | 0.68 ** | 0.05 |  | 0.72 ** | 0.25 ** | 0.48 ** |
| *Merremia hainanensis* | 0.61 ** | 0.17 * |  | 0.41 ** | 0.35 ** | 0.17 * |
| **Lianas** |  |  |  |  |  |  |
| *Podranea ricasoliana* | 0.86 ** | 0.06 |  | 0.68 ** | 0.41 ** | 0.5 ** |
| *Cyclea hypoglauca* | 0.77 ** | 0.42 ** |  | 0.76 ** | 0.04 | 0.44 ** |
| *Tetrastigma planicaule* | 0.69 ** | 0.32 ** |  | 0.32 ** | 0.27 ** | 0.37 ** |

*: *p* <0.05, **: *p* <0.01
